# Supplementary material for: Prevalence of Diarrhoeagenic Escherichia coli among Children Aged between 0–36 Months in Peri-Urban Areas of Lusaka
Source: Microorganisms. 2023 Nov 17;11(11):2790. doi: 10.3390/microorganisms11112790 (PMC10673189; doi:10.3390/microorganisms11112790)
Supplement: Supplementary file 1 [file microorganisms-11-02790-s001.zip › Pathogen Combination_supplementary file Table S2.pdf]

Table S2. Pathotype combinations in samples with more than one pathotype detected and severity of diarrhoea

**Pathogen Combination**	**Total Number of Co-infections**	**Moderate Diarrhoea**	**Severe Diarrhoea**	**P-value**
	n (%)	n (%)	n (%)	
2 Pathogens				
EPEC + ETEC	37 (24.1)	37 (100.0)	0 (0.0)	0.267
EPEC + EAEC	51 (33.3)	49 (96.1)	2 (3.9)	
EPEC + EIEC	26 (16.9)	26 (100.0)	0 (0.0)	
EPEC + EHEC	0 (0.0)	0 (0.0)	0 (0.0)	
ETEC + EAEC	20 (13.0)	18 (90.0)	2 (10.0)	
ETEC + EIEC	6 (3.9)	6 (100.0)	0 (0.0)	
ETEC + EHEC	1 (0.6)	1 (100.00)	0 (0.0)	
EAEC + EIEC	10 (6.5)	9 (90.00)	1 (10.0)	
EAEC + EHEC	1 (0.6)	1 (100.00)	0 (0.0)	
EIEC + EHEC	1 (0.6)	1 (100.00)	0 (0.0)	
3 Pathogens				
EPEC + EAEC + ETEC	28 (40.5)	27 (96.4)	1 (3.6)	1.000
EPEC + EAEC + EIEC	16 (23.1)	16 (100.0)	0 (0.0)	
EPEC + EAEC + EHEC	0 (0)	0 (0.0)	0 (0.0)	
EPEC + ETEC + EIEC	14 (20.2)	14 (100.0)	0 (0.0)	
EPEC + ETEC + EHEC	0 (0)	0 (0.0)	0 (0.0)	
EPEC + EIEC + EHEC	0 (0)	0 (0.0)	0 (0.0)	
EAEC + ETEC + EIEC	9 (13.0)	9 (100.0)	0 (0.0)	
EAEC + ETEC + EHEC	0 (0)	0 (0.0)	0 (0.0)	
EAEC + EIEC + EHEC	2 (2.8)	2 (100.0)	0 (0.0)	
ETEC + EIEC + EHEC	0 (0)	0 (0.0)	0 (0.0)	
4 Pathogens				
EPEC + EAEC + ETEC + EIEC	14 (100.0)	13 (92.9)	1 (7.1)	-
